# Job vacancies at the European Centre for Disease Prevention and Control

**DOI:** 10.2807/1560-7917.ES.2016.21.44.30384

**Published:** 2016-11-03

**Authors:** Eurosurveillance editorial team

**Affiliations:** 1 European Centre for Disease Prevention and Control (ECDC), Stockholm, Sweden

The European Centre for Disease Prevention and Control (ECDC) invites applications for the following positions:

• Scientific Officer Epidemic Intelligence (SRS)

• Medical Library Specialist (RMC)

The deadline for application is 28 November 2016. For detailed information on the job descriptions, requirements and the application procedure, please visit the ECDC website at: http://ecdc.europa.eu/en/aboutus/jobs/Pages/JobOpportunities.aspx

